# SpotLight Proteomics Identifies Variable Sequences of Blood Antibodies Specific Against Deamidated Human Serum Albumin

**DOI:** 10.1016/j.mcpro.2023.100589

**Published:** 2023-06-09

**Authors:** Jijing Wang, Susanna L. Lundström, Weiqi Lu, Yiqi Huang, Sergey Rodin, Roman A. Zubarev

**Affiliations:** 1 Department of Medical Biochemistry and Biophysics, Karolinska Institutet, Stockholm, Sweden; 2 Department of Ophthalmology, First Affiliated Hospital of Xi’an Jiaotong University, Xi’an, China; 3 Institute of Virology, School of Medicine, Technical University of Munich, Munich, Germany; 4 Department of Surgical Sciences, Uppsala University, Uppsala, Sweden

**Keywords:** deamidation, human serum albumin, antibodies, *de novo* sequencing, proteomics, mass spectrometry

## Abstract

Spontaneous deamidation of asparaginyl residues in proteins, if not repaired or cleared, can set in motion a cascade that leads to deteriorated health. Previously, we have discovered that deamidated human serum albumin (HSA) is elevated in the blood of patients with Alzheimer’s disease and other neurodegenerative diseases, while the level of endogenous antibodies against deamidated HSA is significantly diminished, creating an imbalance between the risk factor and the defense against it. Endogenous antibodies against deamidated proteins are still unexplored. In the current study, we employed the SpotLight proteomics approach to identify novel amino acid sequences in antibodies specific to deamidated HSA. The results provide new insights into the clearance mechanism of deamidated proteins, a possible avenue for prevention of neurodegeneration.

## Highlights

• We purified from healthy blood antibodies specific against deamidated HSA.
• SpotLight proteomics uncovered novel isoaspartate-specific antibody sequences.
• Antibodies against deamidated HSA may be specific to other aged proteins.
• Anti-isoaspartate antibodies may render ways of preventing neurodegeneration.

## Graphical Abstract

**Figure:**
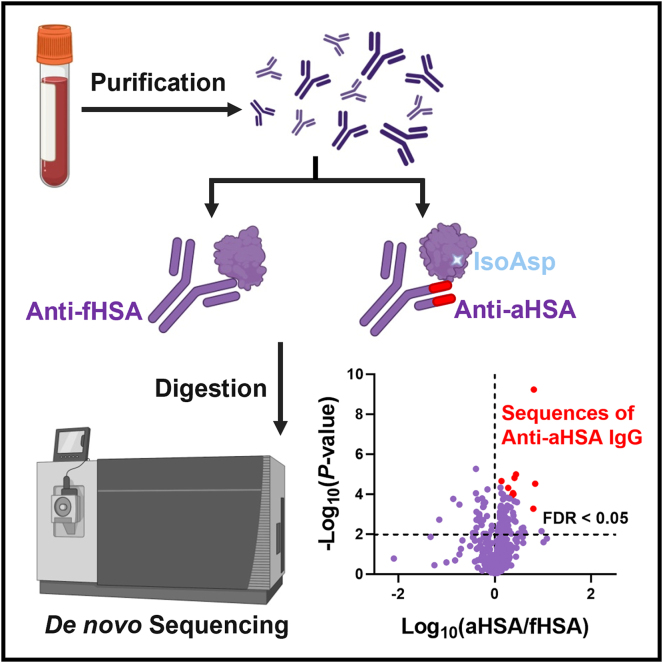

## In Brief

The accumulation of isoaspartate in aged, deamidated blood proteins can lead to deteriorated health, *e.g.*, Alzheimer’s disease. Native antibodies against isoaspartate help maintaining health. We isolated endogenous antibodies against aged human serum albumin and identified novel amino acid sequences in their variable regions. Evidence is found of specificity of these antibodies against other aged proteins. These findings provide new insights into the clearance of aged proteins and indicate a novel possible avenue for prevention and treatment of age-related disorders.

Antibodies (Abs) are proteins produced by the humoral immune system to neutralize pathogens. They recognize the antigen *via* the complementarity-determining regions (CDRs) in the variable fragment antigen-binding (Fab) part of the antibody. To better understand how the acquired immunity works, we need to get a clear picture of how the amino acid sequences of Fab are selected in response to disease or infection.

An old paradigm in immunology posits that the Fab amino acid sequence selection is a completely random process, and thus the probability for two individuals both naive to a given immune challenge of having similar sequences raised in response to it will be vanishingly small (1, 2). This old paradigm is being challenged by the new one postulating that, in a homogeneous group of individuals, the Abs raised in response to a specific challenge to immune system, such as viral infection, will bear similarities in the sequences of the variable regions. This new paradigm has been supported by a number of recent studies (3, 4, 5, 6, 7, 8, 9, 10). Therefore, the fundamental task now pertinent to understanding the human immune system is to assemble a database of specific Fab sequences raised in response to different challenges. This work has already started, and information has been gathered on, for example, Alzheimer’s disease (AD) (11) and sarcoidosis (12) using the so-called SpotLight proteomics approach.

In the SpotLight approach, the Abs are first purified from blood serum or plasma and then digested by trypsin upon S-S bond reduction. The obtained tryptic peptides of the Abs and copurified proteins are separated by liquid chromatography and ionized by electrospray ionization. The ionized molecules are then analyzed by tandem mass spectrometry (MS/MS) employing two complementary fragmentation techniques, high collision dissociation and electron transfer dissociation, which facilitates *de novo* amino acid sequencing (13, 14). The obtained MS/MS spectra are first searched in the database of known sequences, and the unassigned peptides are *de novo* sequenced using appropriate software. Thus, obtained novel sequences that are common for most samples and strongly enriched in the patients compared to healthy controls are flagged as potential “hits”. These hits are further investigated in detail to remove possible false discoveries and compared with similarly obtained sequences from other diseases or infections (11, 12). The validated sequences are then introduced into the database of antibody sequences.

It is of fundamental interest in immunology to understand which Fab sequences are expressed in response to which antigens. Knowing this will allow us to understand better how the human immune system works. One of the important antigens for which the human CDRs are yet unknown is isoaspartyl residue (isoaspartate, isoAsp). IsoAsp is a β-amino acid formed in proteins *via* deamidation of asparaginyl (asparagine, Asn) residue or, less frequently, isomerization of α-aspartyl (aspartate, Asp) residue. Deamidation is a facile, spontaneous, nonenzymatic, and highly damaging posttranslational modification that turns every Asn residue to a potential time bomb inbuilt by nature into every protein (15). Deamidation, if left unchecked, sets a protein aggregation cascade in motion that can ultimately lead to deteriorated health, aging, and neurodegeneration (15, 16, 17, 18). IsoAsp in blood proteins is therefore a risk factor for, for example, AD (19) and other neurodegenerative diseases (20).

Previously, we discovered that deamidation may lead to the aggregation of human serum albumin (HSA), the most abundant protein in blood. Moreover, deamidation reduces the capacity of HSA to bind amyloid-beta (Aβ) peptide and phosphorylated tau (p-tau) protein, which affects Aβ and p-tau clearance enabled *via* HSA transporting them from the brain to the liver and kidneys (19). The reduced clearance of Aβ and p-tau can ultimately cause neurodegeneration (21, 22).

One line of defense against deamidated HSA is its repair by protein L-isoaspartyl methyltransferase enzyme in the liver; another one is the removal by endogeneous polyclonal antibodies (pAbs). However, the level of such pAbs in AD was found to be significantly diminished (15). To test whether this is a common phenomenon in neurodegeneration, we have in a recent study purified immunoglobulins (IgGs) from plasma samples of patients with AD, mild cognitive impairment (MCI), frontotemporal dementia, vascular dementia (VaD), Parkinson’s disease, and healthy controls (total n = 180) (20). Using the artificially aged HSA (aHSA, isoAsp level ∼60%) as an antigen, we have compared the levels of the IgGs against aHSA in these different disease groups with healthy donors. The results have revealed a significant reduction of anti-aHSA IgG levels in patients with AD, MCI, and VaD (*P* < 0.01 in all cases), but possibly not in Parkinson’s disease. These findings supported the notion that the deficit of anti-isoAsp Abs in blood is a risk factor for neurodegeneration and necessitated studying such Abs in greater detail.

One of the anti-isoAsp Abs studied thoroughly is the murine monoclonal antibody of the IgG_3_ type that we have raised against a specific deamidated position in HSA (23). We found that a single amino acid substitution in the heavy and light chain, respectively, can reduce the antibody specificity to isoAsp 1000-fold. Therefore, it is of considerable interest to find isoAsp-specific sequences in endogenous human Abs.

**Fig. 1:**
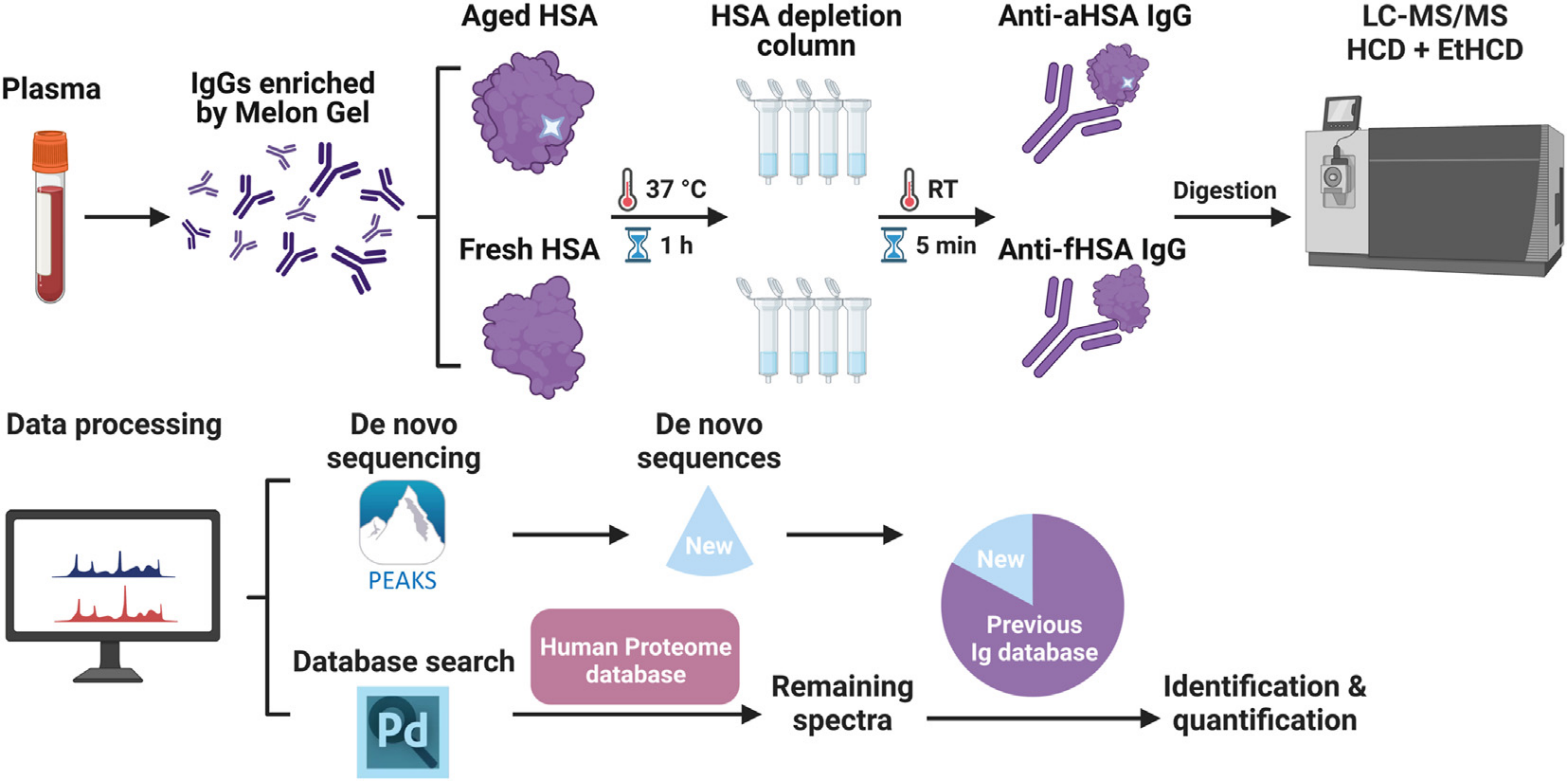
**Schematic overview of the approach**.

Here, we embarked on such a mission with the following approach (Fig. 1). Using artificially deamidated HSA (aHSA) as a bait, we purified specifically binding endogenous Abs from healthy blood extracted from 100 individuals and uncovered the isoAsp-specific sequences with the SpotLight approach. As a control, we used Abs isolated in the same way but using fresh HSA (fHSA) as a bait. Then the unidentified MS/MS spectra were searched against a database containing both *de novo* sequences generated herein and Ig sequences deduced from patients in previous SpotLight studies (11, 12). The obtained isoAsp-specific sequence information can help us better understand the clearance of damaged proteins from blood and thus may show ways of preventing neurodegeneration.

## Experimental Procedures

### Experimental Design and Statistical Rationale

pAbs were purified from blood using Melon Gel, incubated with aHSA and fHSA (control), and the HSA–pAb complexes were isolated using an HSA depletion column. The complexes were then digested with a protease and analyzed by liquid chromatography coupled with tandem mass spectrometry (LC-MS/MS), with database search, *de novo* sequencing, and abundance measurements. Statistically overrepresented peptides for aHSA novel sequences were selected.

### Patients and Samples

Plasma from a cohort of 100 healthy blood donors (age: 36 ± 11 years, female: 41%) was obtained from ProMedDx Limited Liability Company. The samples were collected under a clinical study that had been reviewed by an institutional/independent review board and/or independent ethics committee according to the Declaration of Helsinki principles and the local regulations.

### Artificial Aging of HSA

HSA (Sigma-Aldrich) was incubated in 50 mM Tris buffer (pH 8.0) at 60 °C for 42 days and then reduced, alkylated, digested, and analyzed by LC-MS/MS as described (2). The isoAsp occupancy of all asparagine-containing peptides was determined; the peptide LVNTEFAK showed the highest occupancy (∼60%).

### Purification of IgG from Human Plasma

The IgG Abs were extracted from a pool of 100 healthy human serum samples using the Melon Gel IgG Spin Purification Kit (Thermo Fisher Scientific), with a tandem purification (one after another) performed in four replicates. Then the purified Abs were incubated for 2 h at 37 °C with fHSA and aHSA, respectively, in a 1:1 M ratio. The HSA-IgG complexes were immobilized on the albumin depletion column using the Pierce Albumin Serum Depletion Kit (Thermo Fisher Scientific) following the manufacturer’s protocol. The columns were washed gently three times by the binding buffer, and subsequently the HSA–antibody complexes were eluted from the column by 400 mM NaCl buffer.

### Digestion of Purified HSA–IgG Complexes and Mass Spectrometry Analysis

The eluted HSA-IgG complexes were digested by Lys-C and trypsin (Promega) to peptides and analyzed by LC-MS/MS *via* high collision dissociation combined with electron-transfer/higher-energy collision dissociation fragmentation as in SpotLight analysis (11).

### Proteome Discoverer Database Search and Quantitation

Peptide identification, assembly to proteins, and label-free quantitation were performed using Proteome Discoverer 2.5 software (Thermo Fisher Scientific; https://knowledge1.thermofisher.com/Software_and_Downloads/Chromatography_and_Mass_Spectrometry_Software/Proteome_Discoverer/Proteome_Discoverer_Operator_Manuals/Proteome_Discoverer_2.5_overview). First the MS/MS data were searched against the complete Uniprot human proteome reference database (UP000005640, 20,509 entries) as well as a common contaminant database with reversed sequences concatenated for false discovery rate (FDR) control. The 1% FDR was used as the filter. The unidentified MS/MS spectra were then researched against a database containing Ig sequences (obtained from Uniprot) combined with *de novo*–generated sequences obtained from both the samples herein and previous studies (433 entries, 516,546 residues) (12). The common contaminant database was also included in this second search. Peptide mass error tolerance was set at 10 ppm, while MS/MS fragment mass accuracy was set at 0.02 Da in both searches. Cysteine carbamidomethylation was used as a fixed modification; methionine oxidation, asparagine, and glutamine deamination were used as variable modifications for both identification and quantification. Trypsin was selected as enzyme specificity with maximum of two missed cleavages allowed. One percent FDR was applied as a filter for these data as well. Proteins identified as contaminants (except for serum albumin) as well as reversed protein sequences were removed. Proteins identified by at least one unique peptide and quantified with at least two peptides were kept. Quantification was performed by summing peptide abundances in individual mass spectra over peptide’s chromatographic peaks (*i.e.*, by calculating the areas under the chromatographic curves). Proteins and peptides that were quantified in at least four out of eight samples were kept for analysis. The abundances of Ig peptides and novel peptides were normalized to the total peptide abundance in all eight samples. The abundance of each protein was similarly normalized to the total protein abundance in all eight samples. When a peptide was observed in a given sample below the limit of quantitation range, instead of using zero as its abundance, we used 1/10 of the lowest quantified value (determined for each protein or peptide specifically). Assignment of CDR and framework (FR) regions were based on Uniprot information and by using the VBASE sequence directory (23, 24).

### PEAKs *de Novo* Sequencing

The raw MS/MS data were imported into PEAKS Studio 7.5 (Bioinformatics Solutions Inc), preprocessed *via* precursor mass correction, MS/MS deisotoping and deconvolution, peptide feature detection, and then analyzed to generate a list of peptides. *De novo* sequencing was performed with 10 ppm mass error tolerance for precursors and 0.02 Da for fragment ions. Cysteine carbamidomethylation was set as a fixed modification, and methionine oxidation, asparagine, and glutamine deamidation were marked as variable modifications. The PEAKs *de novo* sequencing score cutoff was set at 70%. The *de novo*–sequenced peptides were incorporated into the Ig sequence database described above.

### Fc-Glycan Profiling

Fc-glycan profiling was performed similarly to what has previously been described (24, 25). Briefly, glycopeptides (from 19 glycoform variants) were identified by their characteristic retention times and accurate monoisotopic masses (within <10 ppm from the theoretical values) of doubly and triply charged ions (IgG_1_: EEQYNSTYR, IgG_2_ or IgG_3_ [IgG_2/3_]: EEQFNSTFR, and IgG_4_ or IgG_3_ [IgG_4/3_]: EEQFNSTYR (or EEQYNSTFR), respectively. Quantification of glycoforms was performed in a label-free manner using Quanti (26). Glycopeptide ion abundances were integrated over respective chromatographic monoisotopic ion peaks (<10 ppm from the theoretical values) at the charged states described above and within a ±1 min interval around the expected retention times. Glycoform abundances were normalized to total content (100%) of Fc-glycosylated IgG_1_ peptides, total content (100%) of Fc-glycosylated IgG_2/3_ peptides, and total content (100%) of Fc-glycosylated IgG_4/3_ peptides, respectively.

### Statistical Analysis

Univariate statistical analysis was performed using two-tailed Student’s *t* test with equal or unequal variance depending upon the F-test, which resulted in *p*-values. FDR was calculated from the *p*-values and the number of tested variables (n = 36 for proteins and n = 536 for the Ig and *de novo*–sequenced peptides) using the Storey Tibshirani method of multiple hypothesis correction. Ig peptides were interrogated both at the 1% and 5% FDR correction level, while 5% FDR correction was set for proteins and peptide deamidation status.

## Results

### Peptide Analysis

In total, 536 peptides originating from Igs or from *de novo*–sequenced peptides with unknown origin were found in the anti-fHSA-IgG and anti-aHSA-IgG pools. After multiple hypothesis correction, *p*-values were converted to FDR. FDR <0.05 and <0.01 were taken as a threshold for statistical significance.

**Fig. 2:**
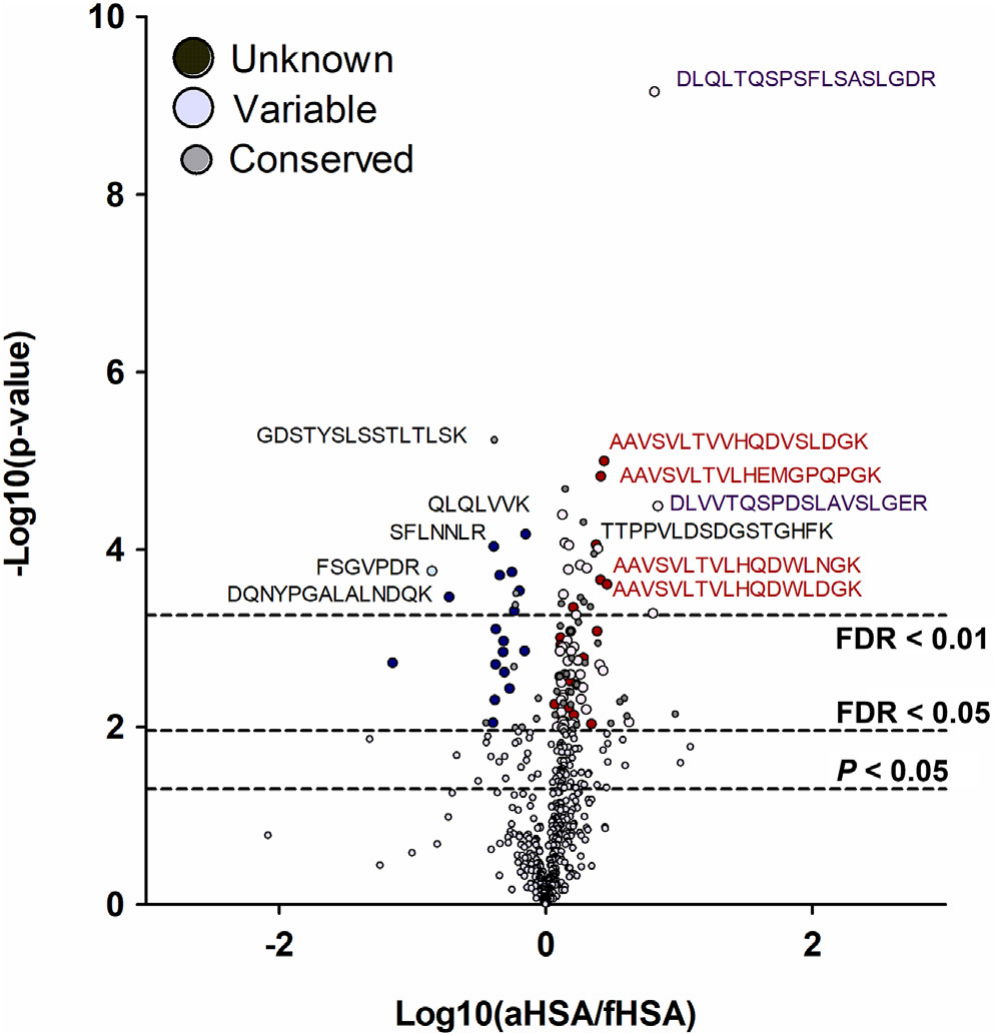
**Volcano plot of the Ig-matching (variable and conserved) and *de novo* sequenced peptides (unknown) with either aHSA specificity (positive x-values) or fHSA specificity (negative x-values).** aHSA, artificially aged human serum albumin; fHSA, fresh human serum albumin.

At 5% FDR, of all peptides, 91 (17%) were significantly overrepresented in terms of their abundances when aHSA was used as a bait compared to just 30 peptides (6%) with fHSA (Table 1 and Fig. 2). Given that fHSA as a normal blood component should not trigger an immune response, while deamidated aHSA is immunogenic, this trend was expected. Notably, among the aHSA-specific peptides were not only Ig-Fab sequences (n = 43) and novel peptides (n = 21) but also sequences obtained from the conserved immunoglobulin regions (n = 27) (Table 1). Importantly, even at a 1% FDR threshold, several enriched peptides formed groups exhibiting sequence similarity (Table 2). A full description of all peptides is given in supplemental Table S1.

**Table 1:** Overview of the Ig-matching and *de novo*–sequenced peptides of unknown origin found in the antibodies binding with fHSA and aHSA. Significance at 5% FDR.

Type	Total found	Significantly enriched in	
		fHSA	aHSA
Conserved	124	11	27
Heavy variable chain	101	0	20
Light variable chain	116	1	23
Unknown	195	18	21
Total	536	30	91

Unknown, *de novo* sequenced peptides with unknown origin.

**Table 2:** Significantly overrepresented peptides at 1% FDR, showing sequence similarity to other such peptides

Enriched with	Sequence	*P*	Log10(aHSA/fHSA)	Origin	Region
fHSA	QLDLNVK	0.005	−0.26	Unknown	−
	QLQLVVK	0.004	−0.15	Unknown	−
aHSA	AAVSVLTVLHEMGPQPGK	0.003	0.41	Unknown	−
	AAVSVLTVVHQDVSLDGK	0.003	0.44	Unknown	−
	AAVSVLTVLHQDWLNGK	0.01	0.41	Unknown	−
	AAVSVLTVLHQDWLDGK	0.01	0.46	Unknown	−
	LSCAASGFTFSSYSMNWVR	0.01	0.28	HV	FR1/CDR1
	LSCAASGFTFDDYAMHWVR	0.005	0.31	HV	FR1/CDR1
	LSCAASGFTFSNAWMSWVR	0.004	0.39	HV	FR1/CDR1
	ATGIPDRFSGSGSGTDFTLTISR	0.01	0.14	KV	FR3
	FSGSGSGTDFTLTLR	0.004	0.14	KV	FR3

CDR, complementarity-determining region; FR, framework region; HV, heavy variable chain; KV, kappa variable chain; region, position in the variable sequence; Unknown, *de novo* sequenced peptides with unknown origin.

### Comparison With SpotLight-Derived Specific Sequences for Neurodegenerative Disorders (DLB and AD) as Well as Sarcoidosis

A total of 117 significant (5% FDR) peptides from variable regions or unknown origin in this study were compared to those in two other Spotlight studies: 322 peptides from study 1 (S1) related to dementia with Lewy bodies (DLB) and with AD (11) and 426 peptides from study 2 (S2) (12) on patients with two types of sarcoidosis and healthy controls. Of the total 865 of such peptides in all three studies, 12 peptides were found in more than one study (Table 3). Notably, all these peptides were found enriched in aHSA complexes. Of these peptides, the most interesting are those originating from the CDR regions, such as the heavy variable chain sequence LSCAASGFTFDDYAMHWVR and the kappa variable chain sequence LLLYSASTLQSGVPSR from CDR1 and CDR2, respectively. Previously the same peptides were found to be significantly elevated in bronchoalveolar lavage of sarcoidosis patients compared to healthy individuals. Also, three of the peptides with unknown origin, YWGQGTLVTVSSASTK, WGQGTLVTVSSASTK, and LLLSWASTR, were both significantly elevated in the sarcoidosis patients. The new sequences WGQGTLVTVSSASTK and LLLSWASTR were also found to be elevated in the AD patients compared to DLB. The annotated MS/MS spectra of sequences listed in Tables 2 and 3 are shown in Supplementary Information.

**Table 3:** Peptides from the variable region or *de novo*–sequenced peptides of unknown origin that were significant in this study (5% FDR) and in at least one other Spotlight proteomics study (S1 and/or S2)

Sequence	Type	Variable region	Significantly elevated in	
			AD or DLB (S1)	S or C
LSCAASGFTFDDYAMHWVR	HV	FR1/CDR1/FR2	−	S (BAL)
AGDTAVYYCAR	HV	FR3	−	S (BAL)
TEDTAVYYCAR	HV	FR3	NS	C (BAL)
AEDTAVYYCAR	HV	FR3	AD (serum)	C (BAL)
LSCAASGFTFSSYGMHWVR	HV	FR1	−	S (BAL)
VEDTAVYFCAR	HV	FR3	NS	C (BAL)
ALQMTQSPSSLSASVGDR	KV	FR1	DLB (MG)	−
DLVMTQSPDSLAVSLGER	KV	FR1	DLB (MG), AD (serum)	NS
LLLYSASTLQSGVPSR	KV	FR2/CDR2/FR3	-	S (BAL)
YWGQGTLVTVSSASTK	Unknown	−	−	S (BAL)
WGQGTLVTVSSASTK	Unknown	−	AD (serum)	S (BAL)
LLLSWASTR	Unknown	−	AD (MG)	S (BAL)

AD, Alzheimer’s disease; BAL, bronchoalveolar lavage; C, Control; CDR, complementarity-determining region; DLB, dementia with Lewy bodies; FR, framework; HV, heavy variable chain; KV, kappa variable chain; MG, Melon Gel enriched; NS, present but not significantly elevated; S, sarcoidosis.

### Protein Abundances

The identified peptides were grouped into proteins when the same protein sequence was identified with at least one unique peptide and quantified with at least two (not necessarily unique) peptides. In total, 36 proteins were quantified this way (supplemental Table S2). As expected, the samples contained mostly Ig heavy constant gamma chains 1 to 4 (IgG_1_-IgG_4_) as well as the light kappa and lambda chains. The samples also contained Ig heavy constant alpha 1 and 2 chains (IgA_1_ and IgA_2_) and as well as immunoglobulin delta heavy chain (IgD). Furthermore, several complement proteins as well as complement activating proteins were observed. Notably, some proteins were found to be significantly enriched in either fHSA or aHSA samples. Most noteworthy, IgA_1_ and complement 3 (one of the key proteins in the complement cascade) had a higher abundance in the aHSA-enriched pAb samples. A full description of all proteins found in the samples is given in supplemental Table S2.

### Deamidated Proteins

**Fig. 3:**
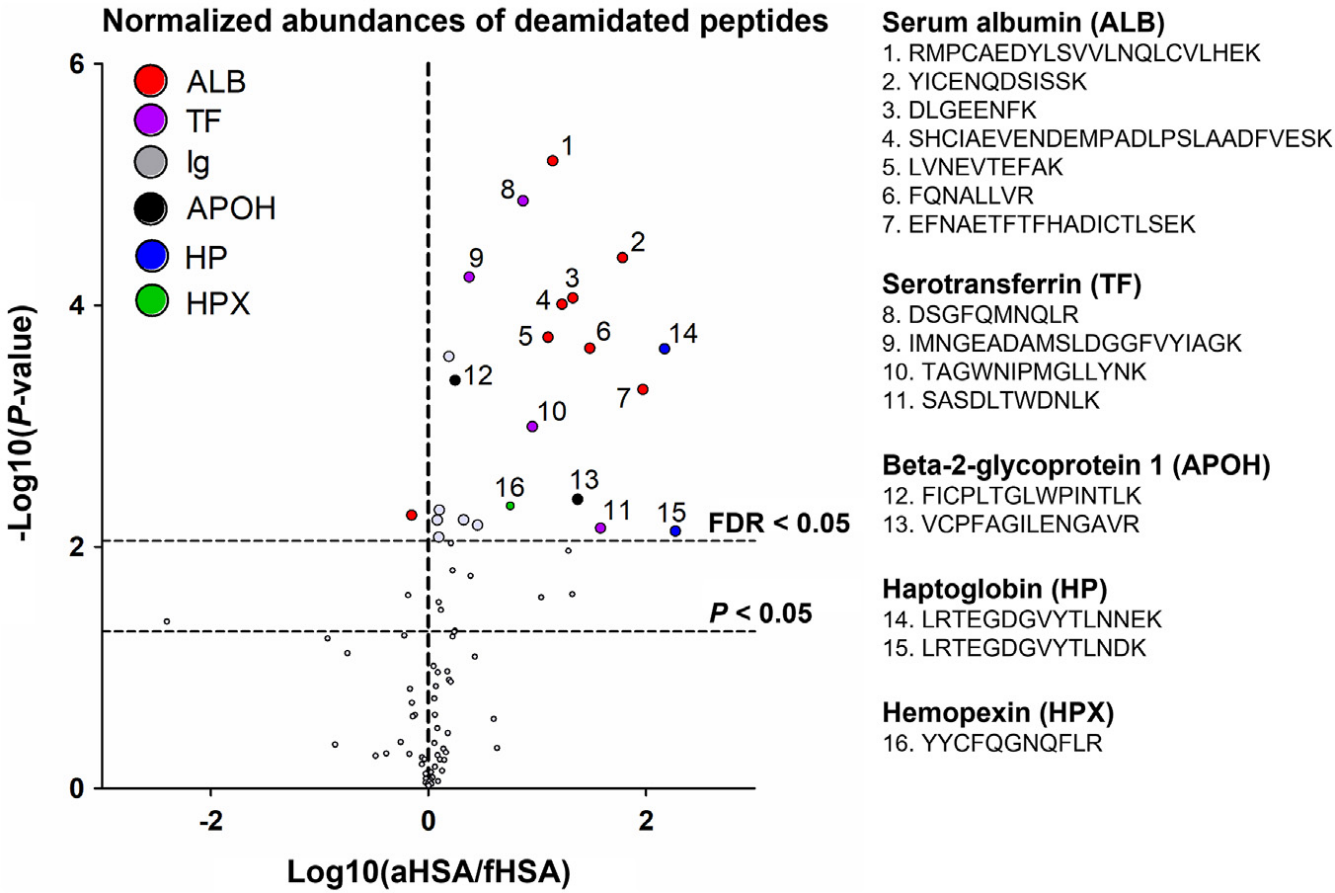
**Volcano plot of the differences in the normalized abundances of deamidated peptides found in the fHSA- and aHSA-enriched pAbs.** pAbs, polyclonal antibodies; aHSA, artificially aged human serum albumin; fHSA, fresh human serum albumin.

Previously, products of asparagine deamidation have been found enriched in AD blood (13). Hence, we included deamidation as a variable modification in the database search. Since deamidation rarely goes to completion, the match was accepted when both the modified and unmodified peptides were found. The abundances of deamidated peptides were first normalized to those of their unmodified counterparts, and thus, obtained deamidation occupancies were compared between the two sample types. The deamidation level was strongly increased in the aHSA-enriched samples, especially in peptides from serotransferrin (TF), beta-2-glycoprotein 1 (APOH), haptoglobin, and hemopexin (Fig. 3). In total, 22 deamidated peptides were significantly enriched in the aHSA samples and only one peptide—in the fHSA samples.

Therefore, it appears that the pAb enriched against aHSA also exhibit specificity to other deamidated proteins. We investigated the position of these deamidation sites in the protein structures and found that a majority were located on the outer surface of the protein and thus are easily accessible for binding with Abs (supplemental Figs. S1–S5). A full description of all deamidated peptides found in the samples is given in supplemental Table S3.

### Fc-Glycosylation Patterns

Previously, we have shown that Fc-glycosylation patterns of pAbs of AD patients differ from those in healthy controls with a lower abundance of complex galactosylated and sialylated forms in AD (20). Now, we investigated whether the IgG Fc-glycosylation pattern would be different between pAbs enriched against fHSA and aHSA. A total of 18 glycopeptides from IgG_1_ (EEQYNSTYR), 11 glycopeptides from IgG_2_ or IgG_3_ (IgG_2/3_, EEQFNSTFR), and 11 glycopeptides from IgG_4_ or IgG_3_ (IgG_4/3_, EEQFNSTYR/EEQYNSTFR) were identified, and their abundances were determined. Overall, there were no distinct significant differences in the glycosylation pattern between the anti-fHSA IgG and anti-aHSA IgG (supplemental Table S4). However, a difference was observed for sialylated forms of the IgG_4/3_ subtype, which were discovered in significantly lower levels (*P* = 0.03) in the anti-aHSA IgG.

## Discussion

In this study, we set out to investigate the sequence differences between the pAbs specific to normal (fresh) HSA compared to deamidated (aged) HSA. Previously, we have found that HSA deamidation leads to aggregation and loss of its carrying function. Importantly, deamidated HSA loses its ability to bind Aβ peptide and p-tau protein, which compromises the removal of these harmful molecules by their transfer from the brain to the liver and kidneys (19). Thus, it has been postulated that an accumulation of deamidated HSA in blood is a risk factor for cognitive decline and neurodegenerative diseases (19). Deamidated HSA and other deamidated blood proteins can be repaired in liver by protein L-isoaspartyl methyltransferase enzyme and are also cleared from the system *via* deamidation-specific Abs. It has been hypothesized that a bottleneck in this clearance process, resulting in accumulation of deamidated proteins in blood, can trigger neurodegeneration. This hypothesis has been strengthened in the recent studies (19, 20) in which the IgG levels of Abs against aHSA were found to be significantly reduced in patients with AD, MCI, and VaD compared to healthy donors.

Little is known regarding human Abs against deamidation. Given that protein deamidation occurs constantly throughout life, it could be hypothesized that these Abs are natural and present in a nonimmunized organism starting from birth, similar to natural antibodies (nAbs) against phosphorylcholine and malondialdehyde (27). Even though nAbs differ in function from adaptive Abs, they tend to be polyreactive and bind to autoantigens and new antigenic determinants (28). Noteworthy, the level of nAbs declines with age, which has been associated with age-related illnesses such as cardiovascular disease and cancer (28, 29). The most common nAb type is IgM, but both IgGs and IgAs can be of importance (30). In this study, IgA_1_ was distinctly enriched for aHSA, consistent with these pAbs to be native.

Furthermore, pAbs enriched against aHSA also reacted with other deamidated blood proteins, such as serotransferrin, beta-2-glycoprotein 1, haptoglobin, and hemopexin. This observation may indicate that the anti-aHSA nAbs are cross-reactive with other deamidated epitopes and could possibly contain pan-isoAsp Abs. The latter is of course a hypothesis that has a low probability, as pan-isoAsp Abs are notoriously difficult to produce. For example, the murine 1A3 monoclonal antibody that we have generated against a specific deamidated site in aHSA did not show any cross-reactivity with other deamidated proteins (16).

Although the majority of the significant new or variable-region peptides were unique to this study compared to two other Spotlight proteomics studies (Table 1 and Fig. 2), some similar peptides were observed, all specific to aHSA (Table 3). In study S1 (11), more common peptides (four) were found elevated in AD than in DLB (two), consistent with HSA deamidation playing role in AD etiology. Among the peptides common with study S2 (12), seven were elevated in sarcoidosis patients compared to three peptides overrepresented in healthy controls. This could be an indicator that deamidated HSA is a factor not only in neurodegeneration but also in sarcoidosis. The cause of the latter disease is unknown but believed to be the body's immune response to an unknown substance. More studies are needed to test the link between deamidation and sarcoidosis. Furthermore, the peptides with no sequence homology to either Igs or other proteins to which we refer in this study as “unknown” should be investigated further to determine if they are indeed CDR3 candidates and/or important for antigen binding.

Given that the fHSA is not immunogenic, the prevalence of aHSA-specific peptides (91 *versus* 30 specific for fHSA) was expected. It is possible that most of the 30 fHSA-enriched peptides are false positives, as the estimate is 27 false positives for 536 peptides detected at FDR = 0.05. If this is the case, the number of true positives in aHSA-enriched sample is close to 60. This result indicates, in line with other studies (25, 27), that the aHSA-enriched pool of pAbs exhibits significant degree of Fab sequence similarity, despite being extracted from the serum of 100 individuals. Therefore, it is not unthinkable that this pool can be separated into several even more homogeneous groups of Abs, perhaps dominated each by a single sequence, with subsequent *de novo* antibody sequencing.

## Conclusion

As *de novo* antibody technology is now relatively mature and accurate (16), it might be possible to derive in this way the sequence of an antibody against aHSA and, possibly, some other deamidated proteins. Such an antibody might have not only analytical potential but also therapeutic prospects in preventing AD and other age-related disorders.

## Data Availability

All data generated or analyzed during this study are included in this publication and/or are available from the corresponding author on reasonable request. The raw MS proteomics data have been deposited to the ProteomeXchange Consortium (http://proteomecentral.proteomexchange.org/) *via* the PRIDE partner repository with the dataset identifier PXD039397.

## Supplemental data

This article contains supplemental data (25).

## Conflict of interest

The authors declare no competing interests.
