# Supplementary material for: SpotLight Proteomics Identifies Variable Sequences of Blood Antibodies Specific Against Deamidated Human Serum Albumin
Source: Mol Cell Proteomics. 2023 Jun 9;22(7):100589. doi: 10.1016/j.mcpro.2023.100589 (PMC10345337; doi:10.1016/j.mcpro.2023.100589)
Supplement: Supplementary information [file mmc1.docx]

**Supplementary information**

**SpotLight proteomics identification of variable sequences in blood antibodies specific against deamidated human serum albumin**

Jijing Wang, Susanna L. Lundström, WeiqiLu, Yiqi Huang, Sergey Rodin, Roman A. Zubarev

**Supplementary Table 1:** List of Ig-peptides and unknown peptides found in the samples – *Excel file*

**Supplementary Table 2:**Proteins found in the samples – *Excel file*

**Supplementary Table 3:** List of the ratio between deamidated peptides/normal peptide in the samples*–Excel file*

**Supplementary Table 4:** Relative abundance of Fc-glycoforms on different IgG-Fc types*–Page 2*

**Supplementary Figure1:**Volcano plot of the proteins found in the samples *–Page 3*

**Supplementary Figure2:** Structure of human serum albumin (HSA) from Protein Data Bank (PDB, ID: 1e78) and positions of identified peptides*–Page 4*

**Supplementary Figure 3:** Structure of serotransferrin (TF) (PDB, ID: 6d04) and positions of identified peptides*–Page 5*

**Supplementary Figure 4:** Structure of Beta-2-glycoprotein 1 (APOH) (PDB, ID: 4jhs) and positions of identified peptides*–Page 6*

**Supplementary Figure 5:**Structure of hemopexin (HPX) (PDB, ID: 1qjs) and position of the identified peptide*–Page 7*

**Supplementary Figure 6:**Structure of haptoglobin-hemoglobin complex (PDB, ID: 4f4o) and position of the identified peptide*–Page 8*

**Supplementary MS/MS Spectra:** MS/MS spectra of sequences listed in Table 2 and Table 3 – *Pages 9-13*

**Supplementary Table 4.**Relative abundance of Fc-glycoformson different IgG-Fc types.

|  | **Fc-glycoform** | **fHSA** | **aHSA** | ***P*** |
| --- | --- | --- | --- | --- |
|  | Gal (-) | 33±12 | 34±9 | 0.8 |
| **IgG_1_** | Gal (+) | 59 ± 9 | 58 ± 6 | 0.9 |
|  | Sia (+) | 8 ± 7 | 8 ± 3 | 0.9 |
|  | Gal (-) | 53 ± 3 | 53 ± 3 | 0.8 |
| **IgG_2/(3)_** | Gal (+) | 47 ± 3 | 47 ± 3 | 0.8 |
|  | Sia (+) | 0.1 ± 0.1 | 0.2 ± 0.2 | 0.2 |
|  | Gal (-) | 59 ± 5 | 52 ± 9 | 0.2 |
| **IgG_4/(3)_** | Gal (+) | 41 ± 5 | 48 ± 9 | 0.2 |
|  | Sia (+) | 3 ± 1 | 2 ± 1 | 0.03 |

Abbreviation: Gal (-), without galactose; Gal (+), with galactose; Sia (+), with sialic acid.

**Reference**

1.Lundstrom, S.L.; Fernandes-Cerqueira, C.; Ytterberg, A.J.; Ossipova, E.; Hensvold, A.H.; Jakobsson, P.J.; Malmstrom, V.; Catrina, A.I.; Klareskog, L.; Lundberg, K.; et al. IgG antibodies to cyclic citrullinated peptides exhibit profiles specific in terms of IgG subclasses, Fc-glycans and a fab-Peptide sequence. PLoS One, 2014,9,e113924.


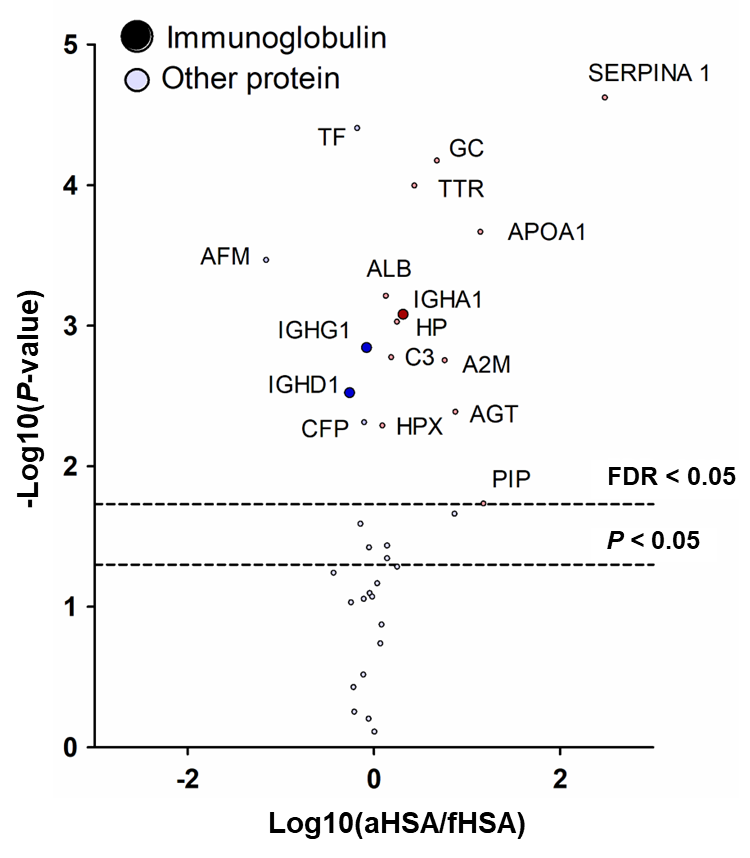


**Supplementary Figure 1:**Volcano plot of the proteins(denoted by their gene names) found in the samples. Proteins enriched in aHSA-specific samplesshow positive Log10(aHSA/fHSA) values.

**
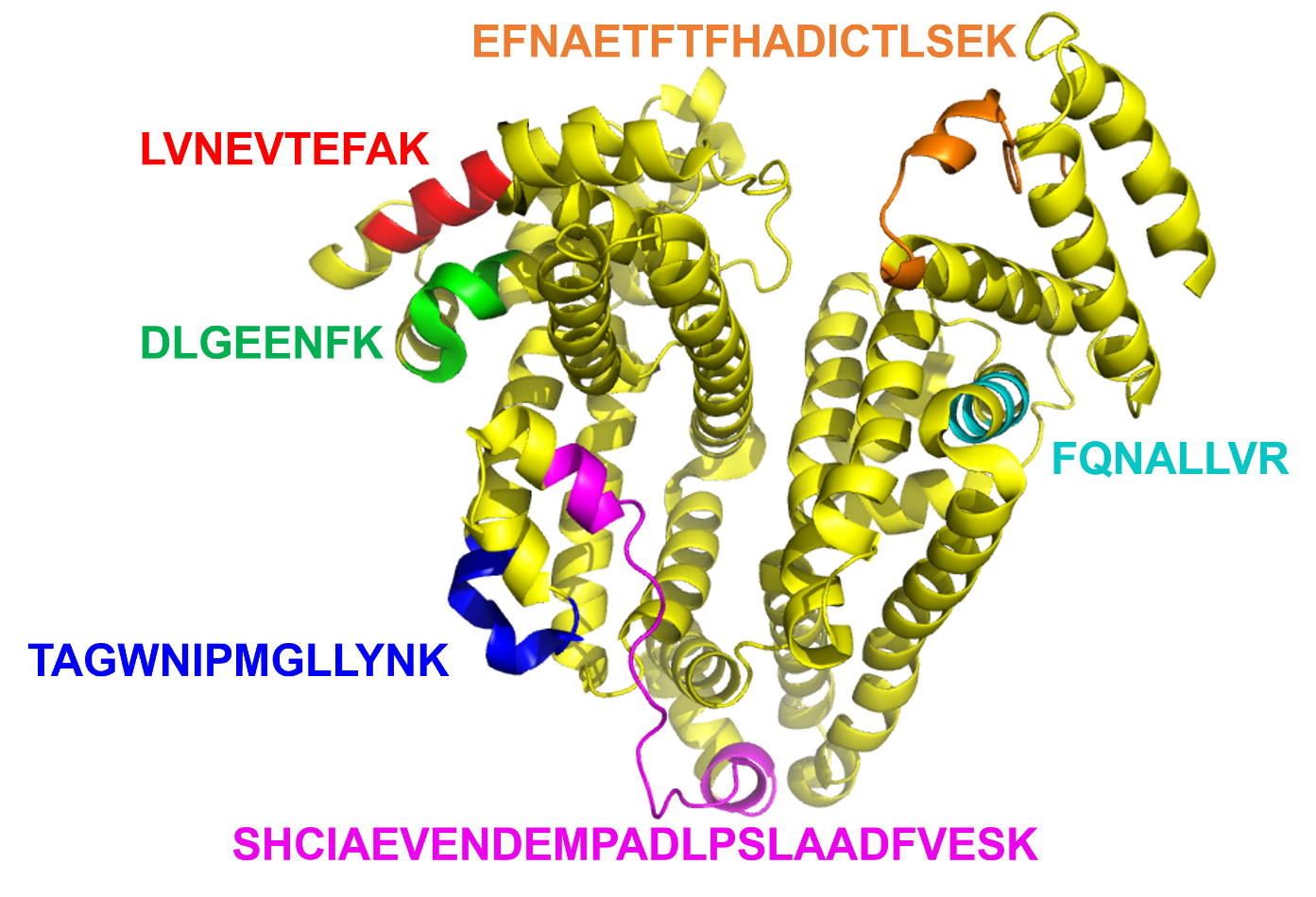
**

**Supplementary Figure 2:** Structure of human serum albumin (HSA) from Protein Data Bank (PDB,ID: 1e78) and positions of identified peptides.

**
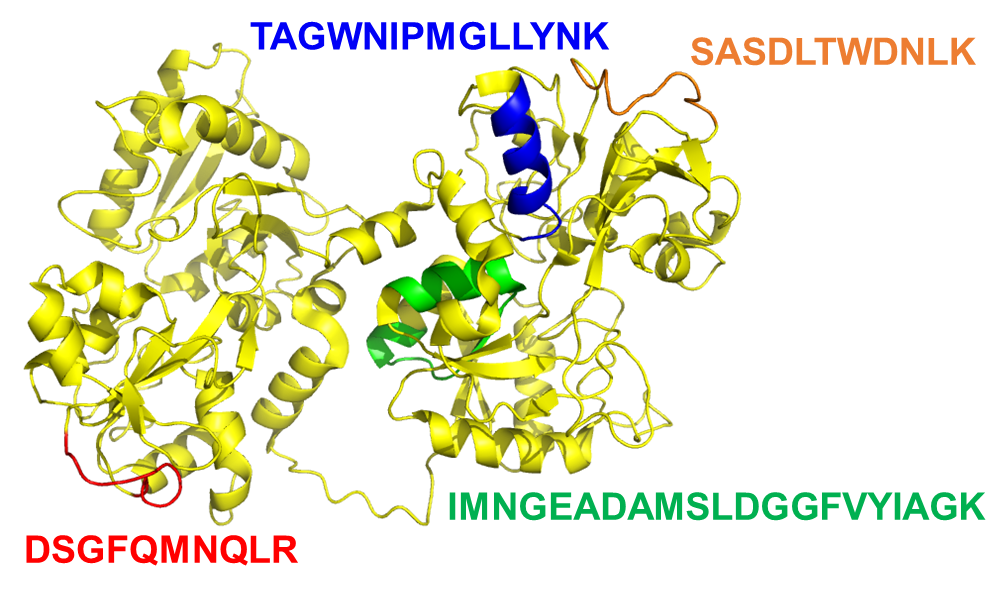
**

**Supplementary Figure 3:** Structure of serotransferrin (TF) (PDB, ID: 6d04) and positions of identified peptides.


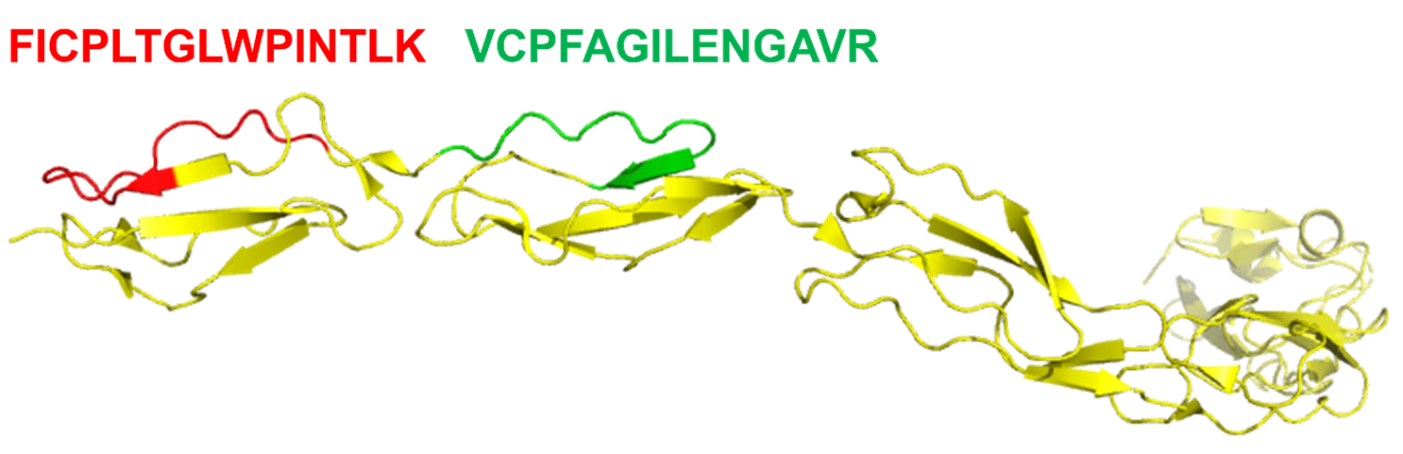


**Supplementary Figure 4:** Structure of Beta-2-glycoprotein 1 (APOH) (PDB, ID: 4jhs) and positions of identified peptides.


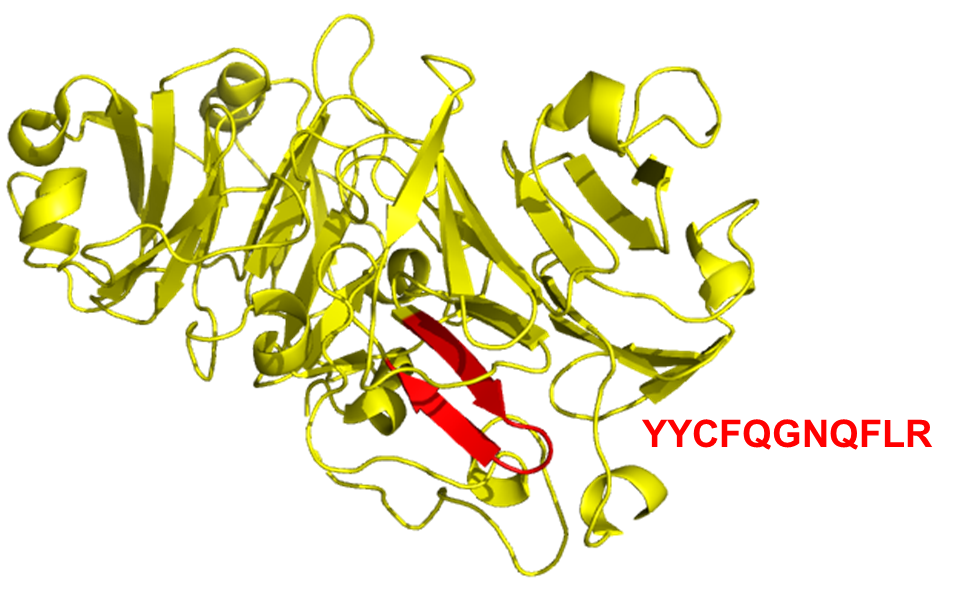


**Supplementary Figure 5:** Structure of hemopexin(HPX) (PDB, ID: 1qjs) and position of the identified peptide.


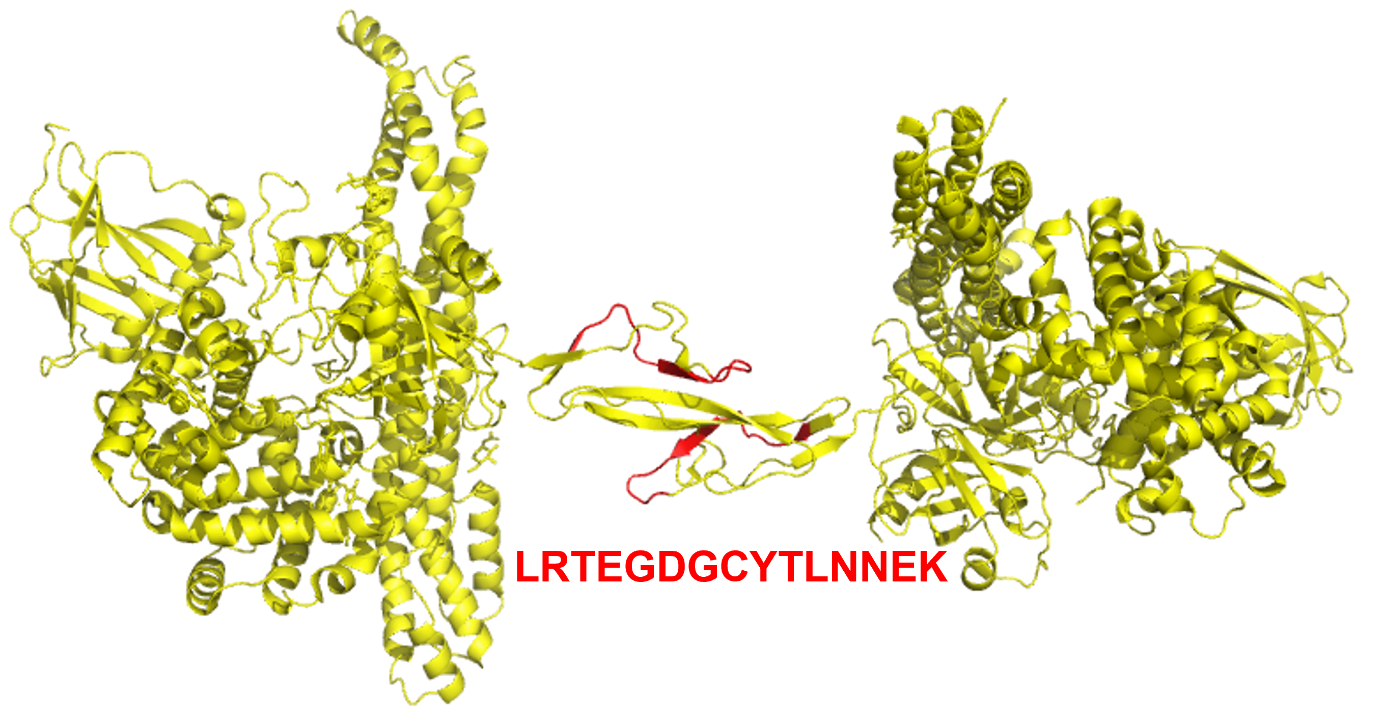


**Supplementary Figure 6:** Structure of haptoglobin-hemoglobin complex (PDB, ID: 4f4o) and position of the identified peptide.

**Supplementary MS/MS Spectra:** MS/MS spectra of sequences listed in **Table 2** and **Table 3**.

Sequences were acquired by first searching the MS/MS data against the complete Uniprot human proteome reference database (UP000005640, 20509 entries) as well as a common contaminant database, with reversed sequences concatenated for 1% FDR control. The unidentified MS/MS spectra were then researched against a database containing Ig sequences (obtained from Uniprot) combined with de novo generated sequences obtained from both the IgG samples herein and previous studies on immunoglobulin (433 entries, 516546 residues). Note that even though this study generated completely new novel sequences, the peptide listed as “unknown” and significant in the aHSA in Table 2 (FDR 1%) and Table 3 (FDR 5%), are reoccurring peptides of unknown origin that were identified in previously sequenced polyclonal IgG.

**Table 2 Spectra:**

**QLDLNVK**

**QLQLVVK**

**AAVSVLTVLHEMGPQPGK**

**AAVSVLTVVHQDVSLDGK**

**AAVSVLTVLHQDWLNGK**

**AAVSVLTVLHQDWLDGK**

**LSCAASGFTFDDYAMHWVR**

(Table 2 and Table 3)

**LSCAASGFTFSNAWMSWVR**

**LSCAASGFTFSSYSMNWVR**

**ATGIPDRFSGSGSGTDFTLTISR**

**FSGSGSGTDFTLTLR**

**Table 3 spectra:**

**LSCAASGFTFSSYGMHWVR**

**AGDTAVYYCAR**

**TEDTAVYYCAR**

**AEDTAVYYCAR**

**VEDTAVYFCAR**

**ALQMTQSPSSLSASVGDR**

**DLVMTQSPDSLAVSLGER**

**LLLYSASTLQSGVPSR**

**YWGQGTLVTVSSASTK**

**WGQGTLVTVSSASTK**

**LLLSWASTR**
